# Supplementary material for: Machine learning outperformed logistic regression classification even with limit sample size: A model to predict pediatric HIV mortality and clinical progression to AIDS
Source: PLoS One. 2022 Oct 14;17(10):e0276116. doi: 10.1371/journal.pone.0276116 (PMC9565414; doi:10.1371/journal.pone.0276116)
Supplement: S1 Table — (DOCX) [file pone.0276116.s001.docx]

**Supplementary Table 1**. Study population characteristics according to the primary outcome (Death/Clinical progression to AIDS)

|  | **Total** | **Did not progress** | **Death/Progressed** | **p-value** |
| --- | --- | --- | --- | --- |
|  | ***N=100*** | ***N=67*** | ***N=33*** |  |
| **Site of recruitment** |  |  |  | 0.002 |
| AHRI | 10 (10.0%) | 7 (10.4%) | 3 (9.1%) |  |
| ARIEL | 12 (12.0%) | 6 (8.96%) | 6 (18.2%) |  |
| CISM | 23 (23.0%) | 9 (13.4%) | 14 (42.4%) |  |
| MALI | 1 (1.00%) | 0 (0.00%) | 1 (3%) |  |
| PHRU | 28 (28.0%) | 23 (34.3%) | 5 (15.2%) |  |
| FAMCRU | 26 (26.0%) | 22 (32.8%) | 4 (12.1%) |  |
| **Age at recruitment** |  |  |  | 0.268 |
| *Months (median, IQR)* | 1.18 [0.98;2.47] | 1.11 [0.77;2.48] | 1.38 [1.05;2.43] |  |
| **Gender** |  |  |  | 0.377 |
| Female | 45 (45%) | 33 (49.3%) | 12 (36.4%) |  |
| Male | 55 (55%) | 34 (50.7%) | 21 (63.6%) |  |
| **Weight at recruitment** |  |  |  |  |
| Weight-for-age (z.score) | -1.44 [-2.68;-0.53] | -1.35 [-2.10;-0.36] | -1.96 [-2.86;-1.17] | 0.050 |
| **Length at recruitment** |  |  |  | 0.628 |
| *Cm (median, IQR)* | 51.8 [48.8;55.0] | 51.5 [48.0;54.0] | 52.0 [49.5;55.0] |  |
| **Preterm birth** |  |  |  | 0.391 |
| No | 62 (62%) | 18 (54.5%) | 44 (65.7%) |  |
| Yes | 38 (38%) | 15 (45.4%) | 23 (34.3%) |  |
| **Age at HIV diagnosis** |  |  |  | 0.002 |
| *Days (median, IQR)* | 30.0 [0.00;35.4] | 1.0 [0.00;32.02] | 33.0 [30.0;48.0] |  |
| **Age at ART initiation** |  |  |  | 0.006 |
| *Months (median, IQR)* | 30.0 [0.00;40.6] | 4.0 [0.00;36.0] | 33.0 [29.0;45.1] |  |
| **Initial ART regimen** |  |  |  | 0.218 |
| 3TC+ABC+LPVr | 47 (47.0%) | 33 (49.3%) | 14 (42.4%) |  |
| 3TC+ABC+NVP | 1 (1.00%) | 1 (1.49%) | 0 (0.00%) |  |
| 3TC+AZT+LPVr | 30 (30.0%) | 16 (23.9%) | 14 (42.4%) |  |
| 3TC+AZT+NVP | 22 (22.0%) | 17 (25.4%) | 5 (15.2%) |  |
| **Pre-ART viral load** |  |  |  | <0.001 |
| *Log10 Copies/mL*  *(median, IQR)* | 5.56 [4.53;6.38] | 5.13 [3.82;6.1] | 6.35 [5.59;6.85] |  |
| **Baseline % CD4** |  |  |  | 0.279 |
| % *Cell/mm^3^ (median, IQR)* | 37.2 [28.0;46.7] | 37.5 [28.2;48.3] | 37.0 [28.0;42.4] |  |
| **Mother’s severe life events or health issues** |  |  |  | 0.362 |
| No | 47 (50.0%) | 31 (46.3%) | 16 (59.3%) |  |
| Yes | 47 (50.0%) | 36 (53.7%) | 11 (40.7%) |  |
| **Mother’s adherence (self-reported at enrollment)** |  |  |  | 0.960 |
| Poor | 5 (6.10%) | 3 (5.45%) | 2 (7.41%) |  |
| Intermediate low | 11 (13.4%) | 7 (12.7%) | 4 (14.8%) |  |
| Intermediate high | 18 (22.0%) | 12 (21.8%) | 6 (22.2%) |  |
| Good | 48 (58.5%) | 33 (60.0%) | 15 (55.6%) |  |
| **Mother’s last CD4 count** |  |  |  | 0.206 |
| *Cell/mL (median, IQR)* | 314 [199;488] | 344 [223;518] | 277 [173;443] |  |
| **Mother’s last viral load count** |  |  |  | 0.467 |
| *Copies/mL (median, IQR)* | 4.5 [3.27;4.97] | 4.47 [3.22;4.93] | 4.53 [3.66; 4.99] |  |
| **Time to follow-up** |  |  |  | <0.001 |
| *Months (median, IQR)* | 12.1 [11.0;16.5] | 16.2 [11.5;16.8] | 5.51 [1.38;11.9] |  |

ART: Antiretroviral; 3TC: Lamivudine; ABC: Abacavir; LPVr: Lopinavir boosted with ritonavir; NVP: Nevirapine; Maternal severe life events: change in employment, separation or relationship break-up, new partner, loss of home or move, or death in the family; Maternal adherence (Optimal: No ART dose missed; Intermediate low: 10-50% doses missed; Intermediate high: 50-90%; Good: >90%). Africa Health Research Institute (AHRI), Durban (South Africa); Family Centre For Research With Ubuntu (FAMCRU), Stellenbosch (South Africa); Perinatal HIV Research Unit (PHRU), Soweto (South Africa); Centro de Investigación en Salud de Manhiça (CISM) – ISGlobal, Manhiça (Mozambique); Ariel Glaser Foundation (ARIEL) (Mozambique).
