# Supplementary material for: Machine learning outperformed logistic regression classification even with limit sample size: A model to predict pediatric HIV mortality and clinical progression to AIDS
Source: PLoS One. 2022 Oct 14;17(10):e0276116. doi: 10.1371/journal.pone.0276116 (PMC9565414; doi:10.1371/journal.pone.0276116)
Supplement: S2 Table — (DOCX) [file pone.0276116.s002.docx]

**Supplementary Table 2**. Feature distribution in the original and imputed data sets

|  | **Training data sets** | | | **Testing datasets** | | |
| --- | --- | --- | --- | --- | --- | --- |
|  | **Original** | **Imputed** | **p-value** | **Original** | **Imputed** | **p-value** |
|  | **N=71** | **N=71** |  | **29** | **29** |  |
| **Age at recruitment** |  |  | 1.00 |  |  | 1.00 |
| Days *(median, IQR)* | 36.0 [0.97;2.26] | 1.18 [0.97;2.26] |  | 1.34 [1.02;2.92] | 1.34 [1.02;2.92] |  |
| **Gender** |  |  | 1.00 |  |  | 1.00 |
| Female | 34 (48.6%) | 35 (49.3%) |  | 11 (37.9%) | 11 (37.9%) |  |
| Male | 36 (51.4%) | 36 (50.7%) |  | 18 (62.1%) | 18 (62.1%) |  |
| **Weight-for-age** |  |  | 1.00 |  |  | 1.00 |
| z-score *(median, IQR)* | -1.47 [-2.62;-0.86] | -1.46 [-2.62;-0.87] |  | -1.18 [-2.98;-0.30] | -1.18 [-2.98;-0.30] |  |
| **Preterm birth** |  |  | 1.00 |  |  | 1.00 |
| No | 40 (57.1%) | 41 (57.7%) |  | 21 (75.0%) | 22 (75.9%) |  |
| Yes | 30 (42.9%) | 30 (42.3%) |  | 7 (25.0%) | 7 (24.1%) |  |
| **Age at HIV diagnosis** |  |  | 1.00 |  |  | 1.00 |
| *Days (median, IQR)* | 30.0 [0.00;35.5] | 30.0 [0.00;35.5] |  | 31.0 [0.00;50.0] | 31.0 [0.00;50.0] |  |
| **Age at ART** |  |  | 0.928 |  |  | 1.00 |
| *Days (median, IQR)* | 32.5 [19.0;62.8] | 32.0 [18.5;62.5] |  | 36.0 [23.0;82.0] | 36.0 [23.0;82.0] |  |
| **Initiation ART regimen** |  |  | 1.00 |  |  | 1.00 |
| 3TC+ABC+LPVr | 33 (46.5%) | 33 (46.5%) |  | 14 (48.3%) | 14 (48.3%) |  |
| 3TC+ABC+NVP | 0 | 0 |  | 1 (3.45%) | 1 (3.45%) |  |
| 3TC+AZT+LPVr | 22 (31.0%) | 22 (31.0%) |  | 8 (27.6%) | 8 (27.6%) |  |
| 3TC+AZT+NVP | 16 (22.5%) | 16 (22.5%) |  | 6 (20.7%) | 6 (20.7%) |  |
| **Baseline viral load** |  |  | 0.920 |  |  | 1.00 |
| Copies/mL *(median, IQR)* | 391854 [30401;2454708] | 609715 [36738;2570245] |  | 226844 [36295;1344319] | 226844 [36295;1344319] |  |
| **Baseline % CD4** |  |  | 0.973 |  |  | 0.886 |
| Cell/mL *(median, IQR)* | 37.0 [28.9;45.4] | 36.9 [29.9;45.2] |  | 38.6 [27.8;46.8] | 40.0 [28.0;47.0] |  |
| **Maternal severe life events or health issues** |  |  | 0.939 |  |  | 1.00 |
| No | 33 (50.0%) | 34 (47.9%) |  | 14 (50.0%) | 14 (48.3%) |  |
| Yes | 33 (50.0%) | 37 (52.1%) |  | 14 (50.0%) | 15 (51.7%) |  |
| **Maternal adherence (self-reported at enrollment)** |  |  | 0.955 |  |  | 1.00 |
| Poor | 3 (4.48%) | 3 (4.23%) |  | 3 (10.7%) | 3 (10.3%) |  |
| Intermediate low | 9 (13.4%) | 12 (16.9%) |  | 3 (10.7%) | 4 (13.8%) |  |
| Intermediate high | 17 (25.4%) | 18 (25.4%) |  | 5 (17.9%) | 5 (17.2%) |  |
| Good | 38 (56.7%) | 38 (53.5%) |  | 17 (60.7%) | 17 (58.6%) |  |
| **Death/Clinical progression to AIDS** |  |  | 1.000 |  |  | 1.00 |
| No | 67 (67.0%) | 67 (67.0%) |  | 9 (31.0%) | 9 (31.0%) |  |
| Yes | 33 (33.0%) | 33 (33.0%) |  | 20 (69.0%) | 20 (69.0%) |  |

ART: Antiretroviral; 3TC: Lamivudine; ABC: Abacavir; LPVr: Lopinavir boosted with ritonavir; NVP: Nevirapine; Maternal severe life events: change in employment, separation or relationship break-up, new partner, loss of home or move, or death in the family; Maternal adherence (Optimal: No ART dose missed; Intermediate low: 10-50% doses missed; Intermediate high: 50-90%; Good: >90%
