# Supplementary material for: Machine learning outperformed logistic regression classification even with limit sample size: A model to predict pediatric HIV mortality and clinical progression to AIDS
Source: PLoS One. 2022 Oct 14;17(10):e0276116. doi: 10.1371/journal.pone.0276116 (PMC9565414; doi:10.1371/journal.pone.0276116)
Supplement: S3 Table — (DOCX) [file pone.0276116.s003.docx]

**Supplementary Table 3**. Model performance in the different subsets

|  |  | **Logistic Regression** | **Random Forest** | **Support Vector Machine** | **Naïve Bayes** | **K-nearest neighbor** | **Artificial Neural Network** | **GLMNet** |
| --- | --- | --- | --- | --- | --- | --- | --- | --- |
| **Real data** | Accuracy | 68 (46.5-85.1) | 76 (54.9-90.64) | 72 (50.1-87.93) | 56 (34.9-75.6) | 72 (50.81-87.93) | 64 (42.5-82.03) | 56 (34.9-75.6) |
|  | Sensitivity | 50 (15.7-84.3) | 75 (34.9-96.81) | 75 (34.91-96.8) | 50 (17.7-84.3) | 75 (34.81-96.8) | 75 (34.9-96.8) | 37.5 (8.5-75.5) |
|  | Specificity | 76.5 (50.2-93.2) | 76.47 (50.1-93.19) | 70.6 (44.04-89.7) | 58.8 (32.9-81.6) | 70.6 (44.0-89.7) | 58.8 (32.9-81.6) | 64.7 (38.3-85.8) |
|  | PPV | 50 (24.93-75.07) | 60 (36.8-79.4) | 54.5 (34.2-73.5) | 36.4 (18.9-58.3) | 54.5 (34.2-73.5) | 46.5 (29.9-63.2) | 33.3 (14.2-60.1) |
|  | NPV | 76.47 (60.8-87.2) | 86.7 (65.5-95.7) | 85.7 (63.5-95.4) | 71.4 (52.9-84.7) | 85.7 (63.5-95.4) | 83.3 (58.5-94.7) | 68.7 (53.7-80.7) |
|  | kappa | 0.26 | 0.48 | 0.41 | 0.08 | 0.42 | 0.29 | 0.02 |
| **A1** | Accuracy | 86.2 (68.3-96.1) | 93.1 (77.2-99.2) | 89.7 (72.6-97.8) | 75.9 (56.4-89.7) | 79.3 (60.3-92.0) | 58.6 (38.9-76.5) | 89.6 (72.6-97.8) |
|  | Sensitivity | 84.6 (54.5-98.1) | 92.3 (69.8-99.8) | 76.9 (46.2-94.9) | 61.5 (31.6-86.1) | 84.6 (54.5-98.1) | 69.2 (38.6-90.9) | 84.6 (54.5-98.1) |
|  | Specificity | 87.5 (61.6-98.4) | 93.7 (69.8-99.8) | 100 (79.4-100) | 87.0 (61.6-98.5) | 75.0 (47.6-92.7) | 50.0 (24.6-75.3) | 93.7 (69.8-99.8) |
|  | PPV | 84.6 (59.6-95.4) | 92.3 (61.1-98.8) | 100 (-) | 80.0 (50.5-94.0) | 73.3 (53.3-86.9) | 52.9 (38.0-67.4) | 91.7 (61.9-98.7) |
|  | NPV | 87.5 (65.9-96.2) | 93.7 (64.1-98.8) | 84.2 (66.4-93.5) | 73.7 (57.8-85.1) | 85.7 (61.9-95.7) | 66.7 (43.6-83.8) | 88.2 (67.6-96.4) |
|  | kappa | 0.72 | 0.86 | 0.79 | 0.50 | 0.59 | 0.19 | 0.79 |
| **A2** | Accuracy | 72.4 (52.7-87.3) | 96.5 (82.2-99.9) | 89.7 (72.6-97.8) | 65.5 (45.7-82.1) | 79.3 (60.3-92.1) | 79.3 (60.3-92.0) | 75.9 (56.5-89.7) |
|  | Sensitivity | 100 (73.5-100) | 100 (73.5-100) | 91.7 (61.5-99.8) | 50 (21.1-78.9) | 91.7 (61.5-99.8) | 91.7 (61.5-99.8) | 100 (73.5-100) |
|  | Specificity | 52.9 (27.8-77.0) | 94.1 (71.3-99.9) | 88.2 (63.6-98.5) | 76.5 (50.1-93.2) | 70.6 (44.0-89.7) | 70.6 (44.0-89.7) | 58.8 (32.9-81.6) |
|  | PPV | 60.0 (47.5-71.3) | 92.3 (64.2-98.8) | 84.6 (59.7-95.3) | 60.0 (34.9-80.7) | 68.7 (50.8-82.4) | 68.7 (50.8-82.4) | 63.2 (49.3-75.2) |
|  | NPV | 100 (-) | 100 (-) | 93.8 (69.5-99.0) | 68.4 (53.7-80.2) | 92.3 (64.2-98.8) | 92.3 (64.2-98.8) | 100 (-) |
|  | kappa | 0.48 | 0.93 | 0.90 | 0.27 | 0.59 | 0.59 | 0.54 |
| **A3** | Accuracy | 86.2 (68.3-96.1) | 93.1 (77.2-99.2) | 72.41 (52.8-87.3) | 68.9 (49.2-84.7) | 93.1 (77.2-99.2) | 79.3 (60.3-92.0) | 89.6 (72.6-97.8) |
|  | Sensitivity | 90.9 (58.7-99.8) | 100 (71.5-100) | 81.8 (48.2-97.7) | 72.7 (39.0-93.9) | 100 (71.5-100) | 100 (71.5-100) | 100 (71.5-100) |
|  | Specificity | 94.4 (58.6-96.4) | 88.9 (65.3-98.6) | 66.7 (40.9-86.7) | 66.7 (41.0-86.7) | 88.9 (65.3-98.6) | 66.7 (41.0-86.7) | 83.3 (58.6-96.4) |
|  | PPV | 90.9 (53.8-90.5) | 84.6 (59.8-95.3) | 60.0 (42.4-75.3) | 57.1 (38.7-73.8) | 84.6 (59.8-95.3) | 64.7 (48.8-77.9) | 78.6 (56.6-91.2) |
|  | NPV | 94.4 (69.6-98.9) | 100 (-) | 85.7 (61.2-95.6) | 80.0 (59.1-91.7) | 100 (-) | 100 (-) | 100 (-) |
|  | kappa | 0.85 | 0.86 | 0.45 | 0.37 | 0.86 | 0.60 | 0.79 |

Real data: data from 100 patients enrolled in the study. A1: Synthetic data resulted from the first resampling after data augmentation (n=100) . A2 Synthetic data resulted from the second resampling after data augmentation (n=100). A3: Synthetic data resulted from the third resampling after data augmentation
