# Supplementary figures and images for: Machine learning outperformed logistic regression classification even with limit sample size: A model to predict pediatric HIV mortality and clinical progression to AIDS
Source: PLoS One. 2022 Oct 14;17(10):e0276116. doi: 10.1371/journal.pone.0276116 (PMC9565414; doi:10.1371/journal.pone.0276116)

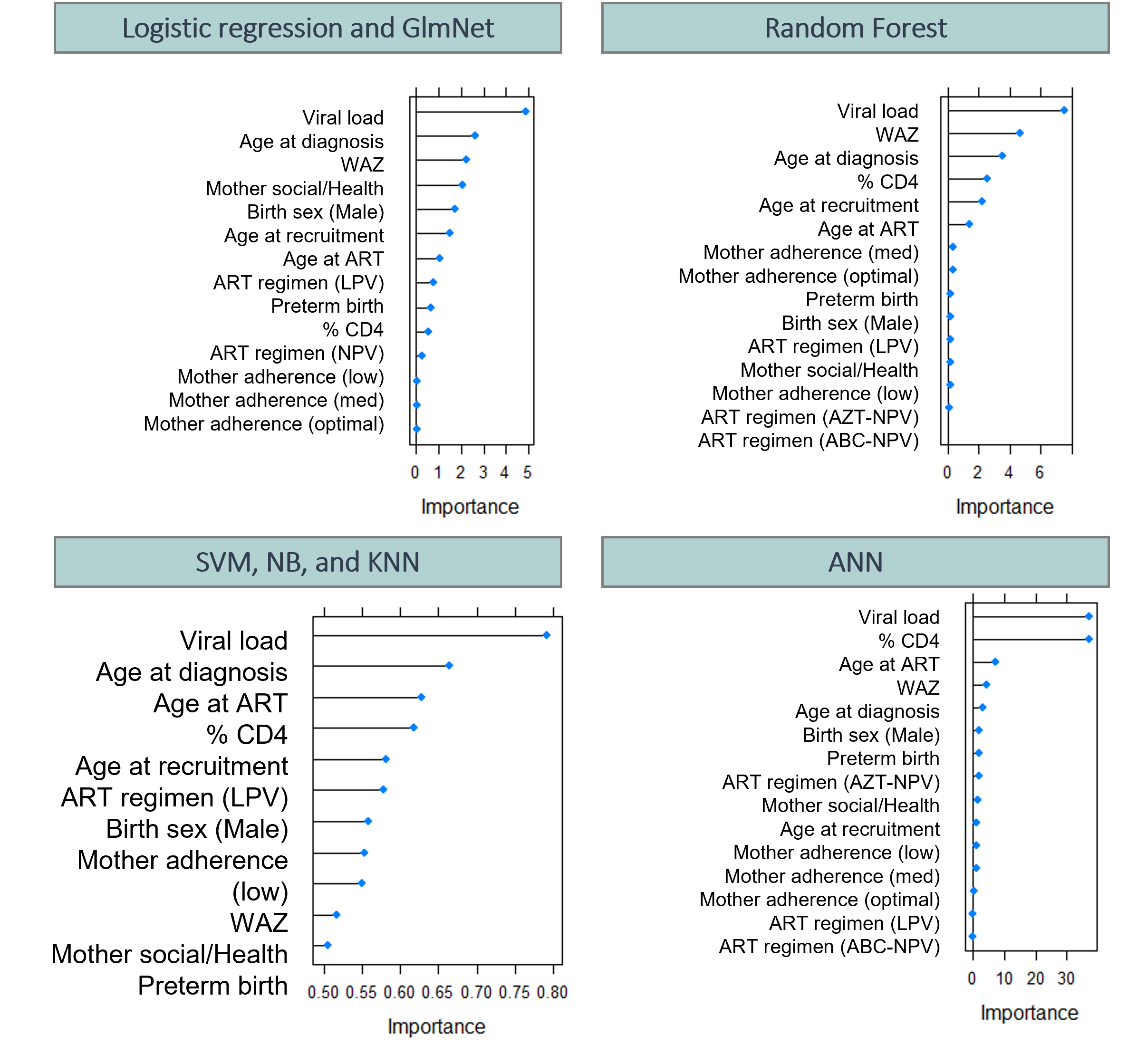

Supplement: S1 Fig — (TIF) [file pone.0276116.s004.tif]
