# Supplementary material for: Machine learning outperformed logistic regression classification even with limit sample size: A model to predict pediatric HIV mortality and clinical progression to AIDS
Source: PLoS One. 2022 Oct 14;17(10):e0276116. doi: 10.1371/journal.pone.0276116 (PMC9565414; doi:10.1371/journal.pone.0276116)
Supplement: Attachment — Submitted filename: Rebuttal_letter_PLOSONE_28-02-22.docx [file pone.0276116.s006.docx]

Alfredo Tagarro

Fundación para la Investigación Biomédica del Hospital 12 de Octubre

Avda. Córdoba, s/n

28041, Madrid, Spain

Phone +34 661810882

alfredotagarro@gmail.com

REBUTTAL LETTER PONE-D-21-31247

**Machine learning outperformed logistic regression classification even with limit sample size: A model to predict pediatric HIV mortality and clinical progression to AIDS**

25^th^ January 2022

Dear editors, please find below the responses in bluish color to each suggestion/comment of the reviewers. Please kindly also find attached in the submission site the manuscript with track changes.

**Editor comments: Journal requirements**

1. Please ensure that your manuscript meets PLOS ONE's style requirements, including those for file naming. The PLOS ONE style templates can be found at
   <https://journals.plos.org/plosone/s/file?id=wjVg/PLOSOne_formatting_sample_main_body.pdf> and 
   <https://journals.plos.org/plosone/s/file?id=ba62/PLOSOne_formatting_sample_title_authors_affiliations.pdf>

We have reformatted the manuscript according to formatting guidelines

2. Please include a complete copy of PLOS’ questionnaire on inclusivity in global research in your revised manuscript. Our policy for research in this area aims to improve transparency in the reporting of research performed outside of researchers’ own country or community. The policy applies to researchers who have travelled to a different country to conduct research, research with Indigenous populations or their lands, and research on cultural artefacts. The questionnaire can also be requested at the journal’s discretion for any other submissions, even if these conditions are not met.  Please find more information on the policy and a link to download a blank copy of the questionnaire here: <https://journals.plos.org/plosone/s/best-practices-in-research-reporting>. Please upload a completed version of your questionnaire as Supporting Information when you resubmit your manuscript. A copy of PLOS questionnaire on inclusivity in global research has been attached.
3. Please provide additional details regarding participant consent. In the ethics statement in the Methods and online submission information, please ensure that you have specified what type you obtained (for instance, written or verbal, and if verbal, how it was documented and witnessed). If your study included minors, state whether you obtained consent from parents or guardians. If the need for consent was waived by the ethics committee, please include this information.

We have included additional information about participant informed consent in the Methods section, study population subsection.

4. We note that the grant information you provided in the ‘Funding Information’ and ‘Financial Disclosure’ sections do not match. We have included the funder ViiV Healthcare in the cover letter
5. We note that you have provided additional information within the Acknowledgements Section that is not currently declared in your Funding Statement. Please note that funding information should not appear in the Acknowledgments section or other areas of your manuscript. We will only publish funding information present in the Funding Statement section of the online submission form.
   Please remove any funding-related text from the manuscript and let us know how you would like to update your Funding Statement. Currently, your Funding Statement reads as follows:

The funders had no role in study design, data collection and analysis, decision to publish, or preparation of the manuscript.

Please include your amended statements within your cover letter; we will change the online submission form on your behalf.

We have included the funding information in the cover letter and remove the funding statement from the acknowledgements section.

6. Please note that in order to use the direct billing option the corresponding author must be affiliated with the chosen institute. Please either amend your manuscript to change the affiliation or corresponding author, or email us at [plosone@plos.org](mailto:plosone@plos.org) with a request to remove this option. The affiliation of the corresponding author has been modified in the manuscript
7. We note that you have stated that you will provide repository information for your data at acceptance. Should your manuscript be accepted for publication, we will hold it until you provide the relevant accession numbers or DOIs necessary to access your data. If you wish to make changes to your Data Availability statement, please describe these changes in your cover letter and we will update your Data Availability statement to reflect the information you provide. The data availability statement has been included in the cover letter.
8. PLOS requires an ORCID iD for the corresponding author in Editorial Manager on papers submitted after December 6th, 2016. Please ensure that you have an ORCID iD and that it is validated in Editorial Manager. To do this, go to ‘Update my Information’ (in the upper left-hand corner of the main menu), and click on the Fetch/Validate link next to the ORCID field. This will take you to the ORCID site and allow you to create a new iD or authenticate a pre-existing iD in Editorial Manager. Please see the following video for instructions on linking an ORCID iD to your Editorial Manager account: <https://www.youtube.com/watch?v=_xcclfuvtxQ>

The ORCID ID from the corresponding author has been updated

9. One of the noted authors is a group or consortium [EPIICAL Consortium]. In addition to naming the author group, please list the individual authors and affiliations within this group in the acknowledgments section of your manuscript. Please also indicate clearly a lead author for this group along with a contact email address.

The members of the EPIICAL consortium have been listed in the Acknowledgements section

**Review Comments to the Author**

Please use the space provided to explain your answers to the questions above. You may also include additional comments for the author, including concerns about dual publication, research ethics, or publication ethics. (Please upload your review as an attachment if it exceeds 20,000 characters)

**Reviewer #1**: Thank you for asking my opinion about the manuscript entitled ‌‎"Machine learning outperformed logistic regression classification even with limit sample size: A model to predict pediatric HIV mortality and clinical progression to AIDS" This manuscript ‎aims to compare the performance of 7 algorithms in the prediction of 1-year mortality and clinical progression to AIDS in a small cohort of infants living with HIV from South Africa and Mozambique.

I believe that this manuscript should ‎be minor revision:‎

The aim of this study (Page 1- Abstract) was there explore compare the performance of 7 algorithms in the prediction of 1-year mortality and clinical progression to AIDS in a small cohort of infants living with HIV from South Africa and Mozambique. There were several good things about the paper, such as ‎aim good. But the abstract should be reformulated and the ‎objective of the study should be well highlighted.‎

1. The abstract is very long.‎

We agree with the reviewer that the abstract was long. However, there is several technical information on methods that take up space and we cannot exclude them. We have now provided an abstract with less than 250 words
‎2. In the introduction, include the significance of the study as ‎well as novelty. What makes the study different from the rest and ‎what does it add to the current knowledge?‎

‎2.1. In the introduction, the authors should have explained the ‎purpose of this study and the existing gaps in this field and ‎explain why this study was conducted.‎

The gaps existing in this fields are detailed in line 18-28 of the introduction where it is explained that despite the advantages on the application of Machine learning stated in previous paragraph, the usability is scarce because the need of large amount of data and lack of interpretabilities models. We have provided line 29-32 explaining the novelty of the study and the aim and discuss the strengths of the study on the discussion section. We have now included in the text that this study presents real-life data instead of simulated data sets.

‎3. In the material and methods section, include sample size justification. ‌‎

We agree with the reviewer that a sample size justification will be interesting for the studies to assess how sample size was chosen. In this study we included a total of 100 children with information available at the moment of the analysis. This information has been newly included in line 48 in the study population subsection of the Material and Methods section. For that reason, there is no sample size calculation. In addition, the required sample size for a particular machine learning model is often unknown. Pre hoc approaches for estimating sample size for machine learning algorithms are known to be not robust in high dimensionality medical data and highly depends on the variability of the sample.

‎4. Conclusions are too short and not supported by results.‎

We have included a more detailed paragraph in the conclusions section of the manuscript

‎5. References are relevant, correct, and not recent. The number of ‎references should be increased.‎

We have included the most recent literature available at the moment. Following the reviewer advice, we have included some more references.

‎6. There are a lot of grammatical errors. This must be taken care ‎of and addressed.‎

We have now corrected grammatical errors and manuscript have been reviewed by a native co-author

**Reviewer #2**:

I find out this article very interesting. It construction is very good and language fulfills scientific research standards.
Selected methods from ML for comparison are general enough and popular enough. I recommend this article for publication.

However I want to authors check if the the sigma value in the SVM method is accurate. If the sigma is very small the SVM tends to make a local classifier, larger sigma tends to make a much more general classifier. Please check if the optimization of this parameter was done right.

We agree with the reviewer in the association between tuning parameters of each model and the results. Also happening with SVM method. In this study we aim to compare the seven methods in predicting the probability of a composite endpoint (death and progression) and we want to follow an off-the-shelf solution without in-house customization that could lead to difficulties in interpreting results or reproducibility. For that reason, we performed an automatic parameter tuning implemented in the caret R package. For each of the novel 6 ML models, a parameter tuning was performed in the training set with 10 tune grids to select the best-performing hyperparameters of each model using 5 times repeated 10-fold cross-validation.

We are aware that, as the reviewer suggested, better models could have been fitted using extensive tuning of the parameters. However, we tried to make it easier and reproducible in the field.

The second thing, which will make this article more clearer to the reader, would be strict references to the Project R packages. I am familiar with this software, but I think that if someone is not, it would be hard to him to reproduce the methods used in results discussion.

We have newly included in the methods section the packages and references for them.

**Reviewer #3:**

The authors compared seven well-understood machine learning (ML) approaches to the problem of predicting mortality and progression to full AIDS on infants with HIV from South Africa and Mozambique. They compared prediction accuracy, sensitivity, and specificity of these methods to highlight the relative benefits of some ML methods and especially against logistic regression, a very common prediction method in medicine. Developing a more accurate predictive model for health outcomes of HIV-infected infants is a very worthwhile task.

Technical Soundness:
The largest obvious challenge with this study, and one that the authors openly recognize, is the small dataset to be used for training and validation. With a 70/30 test-train split on n=100 observations, I believe the authors are challenged to convincingly demonstrate the generalizability of their comparative study. Some ML methods intrinsically perform better than others with smaller training datasets (e.g. Random Forest vs. ANN), but I remain unconvinced the the relative performance of the algorithms described herein would still hold true if one could train on more data. In other words, are the reported relative results really indicative of the true efficacy of the methods or are they significantly influenced by data sparsity alone?
I think it would benefit this comparative study to increase the number of observations for training and validation by at least an order of magnitude. If that can't be done in a practical way with actual observational data due to real-world constraints, I would recommend that the authors look at tabular data augmentation methods to synthetically increase the size of the training and test sets. Tabular data augmentation such as Test-Time augmentation (TTA) can synthetically add new observations by adding small amounts of gaussian noise to duplicated data rows. Libraries such as DeltaPy (<https://github.com/firmai/deltapy>) also assist with meaningful tabular data augmentation and Generative adversarial networks (GANs) and other methods have also been used for this (<https://arxiv.org/abs/2010.00638>). I request the authors to find a way to train and test their algorithms on more data to at least quantify/estimate the effect of small dataset size on the relative performance of the tested results.

We agree with the reviewer that sample size is one of the main limitation of this study. We believe that all of these 7 algorithms, including conventional logistic regression, would benefit from increasing sample size. Our aim was to assess the performance of these 7 algorithms under the same sample size limitation to adjust for real-life research in this setting. We agree that increasing sample size could even make these differences bigger since algorithms such as random forest has already demonstrated better performance in high-dimensional data. The novelty of this approach is to describe and compare the performance of the 7 algorithms under sample size limitation. As the reviewer suggested, we have explored some of the packages for data augmentation such as DeltaPy. However, these approaches are based on data augmentation for deep learning more focused on images classification rather than our aim. In any case, we will explore further types of simulation in next steps and try to find a way to increase sample size based on a sample features distribution.

I would have liked to see some attempt at feature importance estimation under all 7 different algorithms. Which independent variables have the most influence on the balanced accuracy of various ML models? While a bit more challenging for some methods such as ANN, determining relative feature importance under logistic regression and Random Forest is direct and straightforward. Even with ANN, you should be able to do Permutation Feature Importance estimation. Ideally, I would like to see a table of algorithms vs. features/variables ranked by importance. In other words, which features contribute the most to prediction accuracy across the various ML methods? And is there consistency in feature importance across ML methods?
We have now included the Supplementary Figure 1 to describe feature importance across ML the models and the results in Results section.

Figure 2 shows the AUC under the ROC curve and is very telling. An AUC of 50% indicates a totally random classifier, and several of the reviewed algorithms approximated a random classifier with an AUC of close to 50%: Logistic (51.7% AUC), SVM (46% AUC), ANN (52.9% AUC). Really, only Random Forest performed acceptably (73.2%) by the AUC metric, probably because RF had the highest sensitivity. Still, an AUC of 73% is not great. I would love to see how these AUC might change/improve as a function of additional training data.

As mentioned before, we agree with the reviewer that increasing the sample size will improve the performance of each of the models. There are several studies exploring the better performance of ML methods in high dimensional and big data. However, we wanted to compare the performance of these algorithms under the same limitations.

Given the known class imbalance in the data, the authors chose to randomly subsample the already limited training data so that the class frequencies matched the least prevalent class. Another way around this might have been to use a confusion matrix, but report balanced accuracy [(sensitivity + specificity)/2] instead of simple accuracy.

We agree with the reviewer that we could have modified the cut-off that configures the classifier determining an appropriate threshold to balance the accuracy. However we believe that was better to perform downsampling so that physicians and epidemiologist would understand better the sensitivity and specificity result and be more comparable among the ones reported in this setting medical field.

Table 2 shows the tuned hyperparameters discovered by doing a gridded 10-fold cross validation. These are not comprehensive hyperparameters for these algorithms, so it would be helpful to understand how these were chosen. For example, did you only optimize mtry for RF? How did you chose this hyper parameter instead of ntree, sampsize, maxnodes, etc.? Can you include justification for which hyperparameters you chose to optimize for each method?
The hyper parameters shown in Table 2 are the tuning parameters that could be modified and adjust in the caret R package. Tuning parameters are now described in methods section

The data is well-described in the manuscript, but the data is not publicly available. The authors stated that the data is protected by GDPR. I suspect that a sufficiently anonymized version of these data could have been made public in a way that did not conflict with GDPR. If not, please include the actual URL or DOI to the REDCap instance holding these data with better instructions for requesting access.

The study project is still ongoing and database locking has not been performed yet. In addition, this data is allocated and owned by the PENTA Foundation, in Italy and regulated under European GDPR data protection law. According to this regulation, this data is pseudonymized and not anonymized because birth date and site of recruitment is present. Also, there is not a formal consent from the patients or their caregivers to have their data openly available. However, data could be shared under specific circumstances and/or research collaboration by sending a research proposal to the corresponding author of this article.

It would be helpful to review your R source code. Can the authors please publish this source code, perhaps in a public GitHub repository and share the URL or DOI to access to the source code?
We have included the R code in a public repository. The R source code are publicly available at doi:10.5281/zenodo.6303556.

Minor editing: There are various misspellings such as "enrolment" instead of enrollment on lines 46-51. Or "tunning" instead of tuning in Table 2. Lines 181-184 have some english word choice problems such as "These" instead of "This" or "will death" instead of "will die".

We have now corrected grammatical errors and manuscript have been reviewed by a native co-author
