# Supplementary material for: Machine learning outperformed logistic regression classification even with limit sample size: A model to predict pediatric HIV mortality and clinical progression to AIDS
Source: PLoS One. 2022 Oct 14;17(10):e0276116. doi: 10.1371/journal.pone.0276116 (PMC9565414; doi:10.1371/journal.pone.0276116)
Supplement: Attachment — Submitted filename: REVIEWERS_SEP2022.docx [file pone.0276116.s007.docx]

PONE-D-21-31247R1

Machine learning outperformed logistic regression classification even with limit sample size: A model to predict pediatric HIV mortality and clinical progression to AIDS

Please use the space provided to explain your answers to the questions above. You may also include additional comments for the author, including concerns about dual publication, research ethics, or publication ethics. (Please upload your review as an attachment if it exceeds 20,000 characters)

**Reviewer #1**:

Thanks to the corresponding author, he was successful in ‎answering our questions.
Thank you for asking my opinion about the ‎manuscript ‎entitled ‎‎"Machine learning outperformed logistic regression classification even with limit sample size: A model to predict pediatric HIV mortality and clinical progression to AIDS". This ‎manuscript ‎aims to compare the performance of 7 algorithms in the prediction of 1-year mortality and clinical progression to AIDS in a small cohort of infants living with HIV from South Africa and Mozambique. I believe that this manuscript should ‎be ‎minor revision.

No additional requirements were asked by reviewer 1.

**Reviewer #3:**

The authors have revised their manuscript wherein they compared seven well-understood machine learning (ML) approaches to the problem of predicting mortality and progression to full AIDS on infants with HIV from South Africa and Mozambique. The authors should be congratulated for tackling such meaningful and important work.

The authors responded to all reviewer comments and made corrections or additions where possible to address reviewer feedback. The largest problem with this study is the size of the dataset (n=100), as the authors fully recognize. It is not convincing to compare/contrast the performance of machine learning methods against only one extremely small dataset. There is not enough evidence here to demonstrate that Random Forest will generally outperform other methods for these kinds of data. All one can say is that RF outperformed other methods for this single, small dataset. That is an interesting datapoint, but I'm afraid I still remain unconvinced due to lack of sample size and/or replicates. If real-world data was not available (for practical reasons), I would have liked to see some kind of data augmentation via simulation to increase the length of this dataset OR simulate a number of small synthetic n=100 datasets with similar feature distributions. The authors mentioned that their goal was to compare ML methods specifically under sample-size constraints. That is fine, but the investigators should have more replicates (i.e. more n=100 datasets) to provide a convincing case. Comparing these ML methods against real + simulated data would instill more confidence in their comparative study. The authors mentioned that they will seek to explore simulation in future work, but they did not do so for this manuscript. Small sample size with one dataset is still a problem here and the authors have not offered a solution in their response to the reviewers.

I am satisfied that the authors have done feature importance estimation as now mentioned in the results section and supplemental material. I am curious how this estimation was done for methods like ANN, where is it not always clear how features are used in hidden layers. It would be helpful if an extra sentence or two describing how feature importance estimation was done would be helpful. The authors now included Fig 1 in the supplemental material, but I don't have access to that as a reviewer so I cannot comment on that supplemental figure.

The reviewers did a good job at better describing their tunable parameters for all tested algorithms in the Methods section and Table 2. This increases the interpretability and reproducibility of this work.

The authors have made their R code publicly available and have provided a DOI link.

The lack of openly publishing these data may still be an issue. The authors cannot openly and freely share data for a variety of very good reasons, and they have provided alternative methods for investigators to request data on a case-by-case basis. Whether this meets the PLOS ONE standards and requirements for open data is up to the Editor and PLOS ONE.

We appreciate the input and suggestions proposed by the reviewer. We agree with the reviewer that the small sample size could be the largest problem of the study. We also agree that one may think that these results could be biased for these small data sets and appreciate the suggestion of data augmentation creating different small data sets to replicate these results in the same conditions. Using the augmenteR R package we managed to create a oversampled augmented data where we performed resampling with replacement and built three small synthetic dataframes (n=100 each). In each of the synthetic dataframes we performed the same analysis and model comparisons. Similar results were found in the real data and synthetic dataframes with global better results in all the models in the synthetic dataframes (probably because the class imbalance were less). In conclusion, RF was the best fitting model in all the cases validating our previous results. This was added to methods, results, and discussion.
